# Across-sex genomic-assisted genetic correlations for sex-influenced traits in Brahman cattle

**DOI:** 10.1186/s12711-019-0482-6

**Published:** 2019-07-23

**Authors:** Fernanda S. S. Raidan, Laercio R. Porto-Neto, Antonio Reverter

**Affiliations:** 1 CSIRO Agriculture and Food, Castray Esplanade, Battery Point, Hobart, TAS 7004 Australia; 2 CSIRO Agriculture and Food, Queensland Bioscience Precinct, 306 Carmody Rd., St. Lucia, Brisbane, QLD 4067 Australia

## Abstract

**Background:**

This study aimed at estimating genetic parameters of sex-influenced production traits, evaluating the impact of genotype-by-sex interaction, and identifying the selection criteria that could be included in multiple-trait genetic evaluation to increase the rate of genetic improvement in both sexes. To achieve this goal, we used 10 male and 10 female phenotypes, which were measured in a population of 2111 Australian Brahman cattle genotyped at high-density.

**Results:**

Heritability estimates ranged from very low (0.03 ± 0.03 for cows’ days to calving at first calving opportunity, DC1), to moderate (0.33 ± 0.08 for cows’ adult body weight, AWTc), and to high (0.95 ± 0.07 for cows’ hip height, HHc). Genetic correlation (r_g_) estimates between male and female homologous traits were favorable and ranged from moderate to high values, which indicate that selection for any of the traits in one sex would lead to a correlated response with the equivalent phenotype in the other sex. However, the estimated direct response was greater than the indirect response. Moreover, Pearson correlations between estimated breeding values obtained from each sex separately and from female and male homologous traits combined into a single trait in univariate analysis ranged from 0.74 to 0.99, which indicate that small ranking variation might appear if male and female traits are included as single or separate phenotypes. Genetic correlations between male growth and female reproductive traits were not significant, ranging from − 0.07 ± 0.13 to 0.45 ± 0.65. However, selection to improve HHc and AWTc in cows may reduce the percentage of normal sperm at 24 months of age (PNS24), possibly due to correlated effects in the same traits in males, which are related to late maturing animals.

**Conclusions:**

Hip height in cows and PNS24, as well as blood insulin-like growth factor 1 (IGF1) concentration in bulls at 6 months of age are efficient selection criteria to improve male growth and female reproductive traits, simultaneously. In the presence of genotype-by-sex interactions, selection for traits in each sex results in high rates of genetic improvement, however, for the identification of animals with the highest breeding value, data for males and females may be considered a single trait.

**Electronic supplementary material:**

The online version of this article (10.1186/s12711-019-0482-6) contains supplementary material, which is available to authorized users.

## Background

Genetic improvement using breeding techniques such as best linear unbiased prediction of breeding values relies on recording phenotypes. Growth and reproductive traits are two of the most important production traits for cattle breeding systems. On the one hand, growth traits are directly associated to meat, the main sales product of beef cattle [1] and, on the other hand, reproductive traits are a relevant component of economic performance in beef cattle industry [2, 3]. In some cases, these traits are difficult to record or cannot be recorded on the selection candidate, for example, when they are expressed late in life or only in one sex (sex-limited). Furthermore, traits with a low heritability (*h*^2^) are expected to have small rates of genetic improvement. In this situation, indirect selection offers an efficient means of increasing response to selection. For instance, scrotal circumference at a young age, semen quality, and some male hormone levels have been suggested as selection criteria to improve female reproduction traits [4, 5].

Phenotypic means of many traits differ between sexes, and this pattern, which is termed sexual dimorphism, is generally believed to be adaptive [6]. Within-sex variability may have implications for population dynamics, for example, if selection promotes the fixation (or loss) of mutations having sex-limited beneficial (or detrimental) effects [7]. Therefore, if the expression of homologous traits in both sexes is determined to a large extent by different genes, female and male expressions should be treated as separate traits [8]. In scenarios with extreme sexual dimorphism, this could require the implementation of specific selection breeding programs for females and males. Thus, having knowledge about the associations between male and female traits allows us to choose the most efficient selection methods and criteria for better selection decisions. Thus, the aim of this study was to estimate genetic parameters of male and female growth and reproductive traits to guide the identification of selection criteria in multiple-trait genetic evaluation to increase the rate of genetic improvement in both sexes and, for sex-influenced traits, to evaluate the impact of sex-by-genotype interactions on growth and reproductive traits.

## Methods

### Animals, phenotypes and genotypes

Animal Care and Use Committee approval was not obtained for this study because historical data was used and no animals were handled as part of the study. Analyses were performed on phenotypic data and DNA samples that had been collected previously as part of the Cooperative Research Centre for Beef Genetic Technologies (Beef CRC; http://www.beefcrc.com/).

The animals, phenotypes and genotypes that were used in this study were a subset of those controlled by the Cooperative Research Centre for Beef Genetic Technologies northern breeding project. The animals were reared under a range of extensive environments at four research stations in Queensland; details of routine management, health treatments and supplementary feeding regimes are reported in Johnston et al. [9], Burns et al. [10], Wolcott et al. [11] and Porto-Neto et al. [12]. In brief, we used data on 2111 Brahman cows and bulls that were genotyped by using either the BovineSNP50 [13] or the BovineHD (Illumina Inc., San Diego, CA), which includes more than 770,000 single nucleotide polymorphisms (SNPs). Animals that were genotyped with a lower density array had their genotypes imputed to higher density as described by Bolormaa et al. [14]. For the present study, we used 651,253 SNPs mapped to autosomal chromosomes and with a minor allele frequency higher than 5% in the entire population of cows and bulls.

In total, 995 Brahman females and 1116 Brahman males were available for this study. They were measured for a range of reproductive and growth traits including: age at detection of the first corpus luteum (ACL, days); age at an estimated scrotal circumference of 26 cm (AGE26, days); days-to-calve at first calving opportunity, defined by days from start of specified mating season to subsequent calving (DC1, days); days-to-calve averaged for 5 breeding opportunities (DC5, days); blood concentration of IGF1 at 6 (males) and 18 (females) months of age (IGF1, ng/mL); blood concentration of inhibin at 4 months of age in males (IN4, ng/mL); eye muscle area at mature adult age in females and post yearling in males (EMA, cm2); hip height at mature adult age in females and post yearling in males (HH, cm); body weight at mature adult age in females and post yearling in males (AWT, kg); percentage of normal sperm at 24 months of age (PNS24,  %); post-partum anoestrus interval (PPAI, days); scrotal circumference at 12 months of age (SC12, cm); body condition score at yearling (BCS, points 1–10); and body weight at yearling (YWT, kg). Description of all traits, and their means and standard deviations are in Table 1. The dataset included 110 known sires, with an average of 18.8 progeny and a number of progeny ranging from 1 to 57, and 1433 known dams, with an average of 1.4 progeny and a number of progeny ranging from 1 to 7.

**Table 1:** Description of traits, number of records (N), means ± standard deviations, and fixed effects included in the models for the analysis of 20 growth and reproductive traits, 10 for females and 10 for males, in a population of Brahman cattle

Trait	Description	Summary statistics		Fixed effect^a^			
		N	Mean	CG	BATCH	AOD	AGE
ACL	Age at detection of the first corpus luteum (d)	980	750.65 ± 141.80	x			
AGE26	Age at estimated scrotal circumference of 26 cm (d)	1044	554.92 ± 101.11	x			
DC1	Days-to-calving at first calving opportunity (d)	995	345.41 ± 48.49	x			
DC5	Days-to-calving averaged for 5 breeding opportunities (d)	794	344.37 ± 19.23	x			
IGF1b	Bulls’ blood concentration of IGF1 at 6 mo of age (ng/mL)	1051	544.57 ± 325.25	x	x	x	
IGF1c	Cows’ blood concentration of IGF1 at 18 mo of age	995	191.33 ± 89.30	x			x
IN4	Blood concentration of IN4 at 4 mo of age (ng/mL)	786	7.36 ± 1.92	x			
EMAb	Bulls’ eye muscle area at 24 mo of age (cm^2^)	1097	61.69 ± 7.95	x		x	
EMAc	Cows’ eye muscle area at adult age (cm^2^)	920	46.34 ± 7.49	x			
HHb	Bulls’ hip height at yearling (cm)	1097	140.95 ± 4.49	x		x	
HHc	Cows’ hip height at adult age (cm)	914	127.84 ± 4.59	x			
AWTb	Bulls’ body weight at adult age (kg)	1116	499.54 ± 52.48	x		x	x
AWTc	Cows’ body weight at adult age (kg)	923	308.72 ± 38.67	x			x
PNS24	Percent normal sperm at 24 mo of age (%)	964	73.55 ± 22.06	x			
PPAI	Post-partum anoestrus interval (d)	618	180.37 ± 109.05	x			
SC12	Scrotal circumference at 12 mo of age (cm)	1112	21.24 ± 2.72	x			x
BCSb	Bulls’ body condition score at yearling (1–10)	1116	6.70 ± 0.50	x			
BCSc	Cows’ body condition score at yearling (1–10)	995	8.01 ± 0.86	x			
YWTb	Bulls’ body weight at yearling (kg)	1116	243.70 ± 29.18	x		x	x
YWTc	Cows’ body weight at yearling (kg)	995	209.75 ± 30.54	x			x

^a^CG = contemporary group; BATCH = laboratory assay batch; AOD = age of dam; AGE = age of animal at measurement; mo: month

### Statistical analyses

**Fig. 1:**
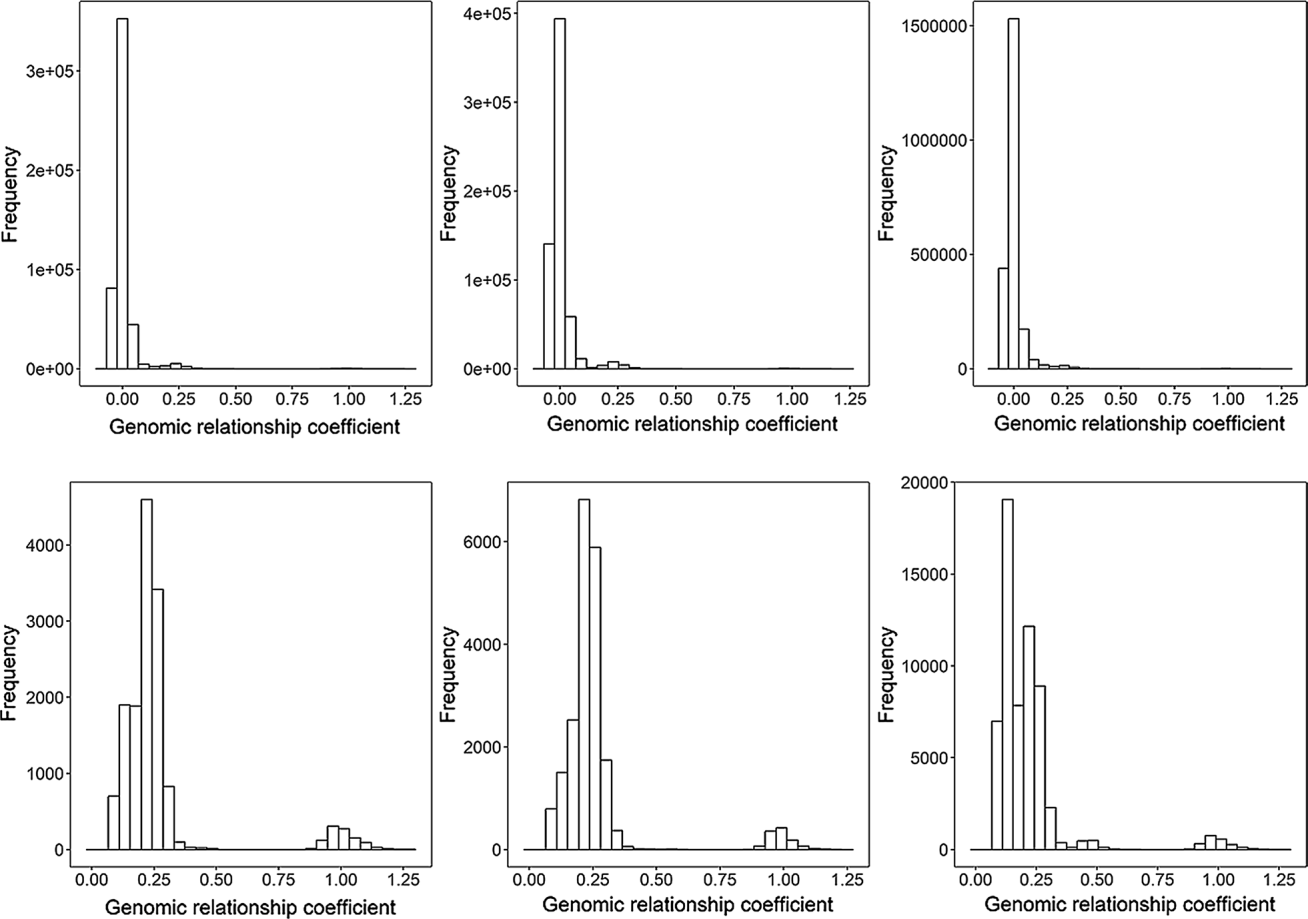
Frequency distribution for genomic relationship coefficients among females (n = 995, left panels), males (n = 1116, middle panels) and all animals (n = 2111, right panels), among 2111 animals (top panels) and among animals with a relationship coefficient higher than 0.1 (bottom panels)

All analyses were carried out using the AIRemlf90 software program [15]. Estimates of variance components for each pair of male and female traits were obtained using the genomic best linear unbiased prediction method in a series of 100 bivariate analyses (i.e. from 10 traits in males and 10 traits in females). In all cases, the same genomic relationship matrix (**G**) was used and computed following Method 1 of VanRaden [16]. The distribution of the genomic relationship coefficients both within and across sexes is shown in Fig. 1.

The following general mixed model [17] was used to estimate variance and covariance components for each pair of traits:

$$\left[ {\begin{array}{*{20}c} {{\mathbf{y}}_{\text{M}} } \\ {{\mathbf{y}}_{\text{F}} } \\ \end{array} } \right] = \left[ {\begin{array}{*{20}c} {{\mathbf{X}}_{\text{M}} } & \emptyset \\ \emptyset & {{\mathbf{X}}_{\text{F}} } \\ \end{array} } \right]\left[ {\begin{array}{*{20}c} {{\varvec{\upbeta}}_{\text{M}} } \\ {{\varvec{\upbeta}}_{\text{F}} } \\ \end{array} } \right] + \left[ {\begin{array}{*{20}c} {{\mathbf{Z}}_{\text{M}} } & \emptyset \\ \emptyset & {{\mathbf{Z}}_{\text{F}} } \\ \end{array} } \right]\left[ {\begin{array}{*{20}c} {{\mathbf{u}}_{\text{M}} } \\ {{\mathbf{u}}_{\text{F}} } \\ \end{array} } \right] + \left[ {\begin{array}{*{20}c} {{\mathbf{e}}_{\text{M}} } \\ {{\mathbf{e}}_{\text{F}} } \\ \end{array} } \right],$$

where ${\mathbf{y}}_{\text{M}}$ and ${\mathbf{y}}_{\text{F}}$ represent the phenotypic observations for males (${\text{M}}$) and females (${\text{F}}$), respectively, ${\mathbf{X}}$ is the incidence matrix relating fixed effects in ${\varvec{\upbeta}}$ with observations in ${\mathbf{y}}_{\text{M}}$ and ${\mathbf{y}}_{\text{F}}$, ${\mathbf{Z}}$ is the incidence matrix that allocates records to breeding values in ${\mathbf{u}}$ for every individual in the relationship matrix (both males and females), and ${\mathbf{e}}_{\text{M}}$ and ${\mathbf{e}}_{\text{F}}$ are the random residual effects for males and females, respectively. The fixed effects included in the model were specific for each evaluated trait and are described in Table 1. The fixed effects of contemporary group (72 levels for females and 60 levels for male), laboratory assay batch (52 levels), age of dam and age of the animal at the time of recording (linear covariable) were considered and included in the model when significant.

The efficiency of the correlated response (ECR) to selection was obtained by:

$${\text{ECR}}_{\text{FM}} = \frac{{\Delta {\text{G}}_{\text{FM}} }}{{\Delta {\text{G}}_{\text{F}} }} = {\text{r}}_{{{\text{a}}_{\text{FM}} }} \frac{{{\text{h}}_{\text{F}} }}{{{\text{h}}_{\text{M}} }},$$

where $\Delta {\text{G}}_{\text{FM}}$ is the expected correlated response per generation relative to a given female trait by selecting for the male trait, $\Delta {\text{G}}_{\text{F}}$ is the expected direct response per generation relative to a given female trait, ${\text{r}}_{{{\text{a}}_{\text{FM}} }}$ represents the genetic correlation of a trait measured in females and males obtained in bivariate analysis, ${\text{h}}_{\text{F}}$ and ${\text{h}}_{\text{M}}$ represent the square root of the heritability *h*^2^ for females and males, respectively.

The efficiency of predictive power of genomic estimated breeding values (GEBV) for the same trait measured in both sexes was determined by the ratio of correlations between the adjusted phenotype for a given sex and GEBV in the same sex or the opposite sex. In other words, for any given trait, two GEBV were obtained, i.e. the female and male GEBV, both of the same dimension and equal to the total number of animals (N = 2111) including 995 females and 1116 males. The correlation between the within-sex GEBV and the adjusted phenotype was computed separately for the animals of each sex.

In addition to the analyses previously described, six single-trait analyses were performed, in which female and male homologous traits were combined into a single trait. These analyses, which included sex and all the other fixed effects cited above, were performed to estimate the genetic parameters and GEBV, and to compare the results from the analyses that treat traits either separately for each sex or combined for both sexes.

## Results

### Heterogeneity of variances and heritability for male and female growth and reproductive traits

Table 2 shows the additive and residual variances, heritability estimates and corresponding standard errors (SE) for all evaluated traits. Values are available for bivariate models in which traits were included for each sex separately, and for univariate analyses in which traits for both sexes are combined into a single trait. For bivariate models, the values presented are the average of 10 estimates obtained for each trait. When estimated separately, male and female genetic parameters were usually different, whereas those obtained for the sex-combined trait had intermediate values. For instance, estimates of additive genetic variance ± SE for BCS were equal to 0.08 ± 0.02 and 0.42 ± 0.07 for males and females, respectively, and 0.20 ± 0.02 for the sex-combined trait (Table 2). In spite of differences in the estimates of variance components, the estimates of the heritability *h*^2^ for male and female traits were similar, except for the HH trait, for which *h*^2^ was higher in females (0.95 ± 0.07) than in males (0.62 ± 0.07).

**Table 2:** Means and standard deviations (SD) for additive ($\sigma_{a}^{2}$) and residual variance ($\sigma_{e}^{2}$) and *h*^2^ estimates for growth and reproductive traits in Brahman cattle obtained across the 6 single-trait and 10 two-trait analyses in which each trait was included

Trait^a^	Two-trait			Single male + female joined		
	$\sigma_{a}^{2}$	$\sigma_{e}^{2}$	*h* ^2^	$\sigma_{a}^{2}$	$\sigma_{e}^{2}$	*h* ^2^
ACL	7733.9 ± 1346.6	5599.7 ± 998.4	0.58 ± 0.08	–	–	–
AGE26	4911.9 ± 712.5	2672.9 ± 467.4	0.65 ± 0.07	–	–	–
DC1	12.4 ± 20.8	436.3 ± 28.1	0.03 ± 0.05	–	–	–
DC5	58.8 ± 20.4	146.6 ± 18.7	0.29 ± 0.09	–	–	–
IGF1b	9251.2 ± 1644.2	8795.1 ± 1177.9	0.51 ± 0.08	3088.2 ± 475.9	7133.8 ± 403.1	0.30 ± 0.04
IGF1c	1121.4 ± 235.5	1444.7 ± 190.2	0.44 ± 0.08			
IN4	2.2 ± 0.6	0.9 ± 0.3	0.71 ± 0.08	–	–	–
EMAb	10.4 ± 2.3	16.9 ± 1.8	0.38 ± 0.08	11.4 ± 1.9	26.3 ± 1.6	0.30 ± 0.06
EMAc	17.7 ± 4.7	32.7 ± 4.0	0.35 ± 0.09			
HHb	8.6 ± 1.3	5.2 ± 0.9	0.62 ± 0.07	12.2 ± 1.0	4.5 ± 0.5	0.73 ± 0.04
HHc	18.6 ± 2.1	0.9 ± 1.4	0.95 ± 0.07			
AWTb	352.9 ± 61.3	303.18 ± 42.8	0.54 ± 0.08	549.1 ± 66.6	644.4 ± 46.3	0.46 ± 0.05
AWTc	1255.5 ± 197.8	602.9 ± 140.2	0.70 ± 0.07			
PNS24	69.7 ± 31.5	449.8 ± 21.9	0.13 ± 0.07	–	–	–
PPAI	4221.5 ± 1184.7	5970.2 ± 1006.8	0.42 ± 0.10	–	–	–
SC12	2.7 ± 0.4	2.1 ± 0.3	0.56 ± 0.07	–	–	–
BCSb	0.08 ± 0.0	0.09 ± 0.0	0.51 ± 0.07	0.2 ± 0.02	0.19 ± 0.01	0.52 ± 0.04
BCSc	0.42 ± 0.1	0.21 ± 0.1	0.66 ± 0.08			
YWTb	244.9 ± 49.3	304.1 ± 34.6	0.45 ± 0.08	160.5 ± 23.6	293.1 ± 18.3	0.35 ± 0.05
YWTc	174.9 ± 36.9	188.4 ± 28.8	0.48 ± 0.09			

^a^Traits are as described in Table 1

In general, *h*^2^ estimates ranged from moderate to high values, which indicates that the means for the traits evaluated can be modified through selection. For growth traits (YWT, AWT, and HH), *h*^2^ estimates ranged from 0.33 ± 0.08 to 0.95 ± 0.07. For EMA, they were moderate in both females (0.35 ± 0.09) and males (0.38 ± 0.08). Higher *h*^2^ estimates were obtained for hormone levels, i.e. 0.41 ± 0.08 for insulin-like growth factor 1 in cows (IGF1c), 0.51 ± 0.08 for IGF1 in bulls (IGF1b) and 0.71 ± 0.08 for IN4 levels. Some female reproductive traits including ACL and PPAI had high *h*^2^ of 0.58 ± 0.08 and 0.42 ± 0.10, respectively, and could be used as selection criteria to improve such traits in females (Table 2). However, for DC1 and DC5, *h*^2^ estimates were much lower at 0.03 ± 0.05 and 0.29 ± 0.09, respectively. In addition, the genetic variability of these traits is limited in Brahman cattle, thus they will respond slowly to selection, especially DC1. For some male reproductive traits (AGE26, SC12 and IN4 levels), *h*^2^ estimates were high with values higher than 0.56 ± 0.07, whereas for PNS24, *h*^2^ was low, i.e. 0.13 ± 0.07 (Table 2).

### Across-sex genetic analyses

**Fig. 2:**
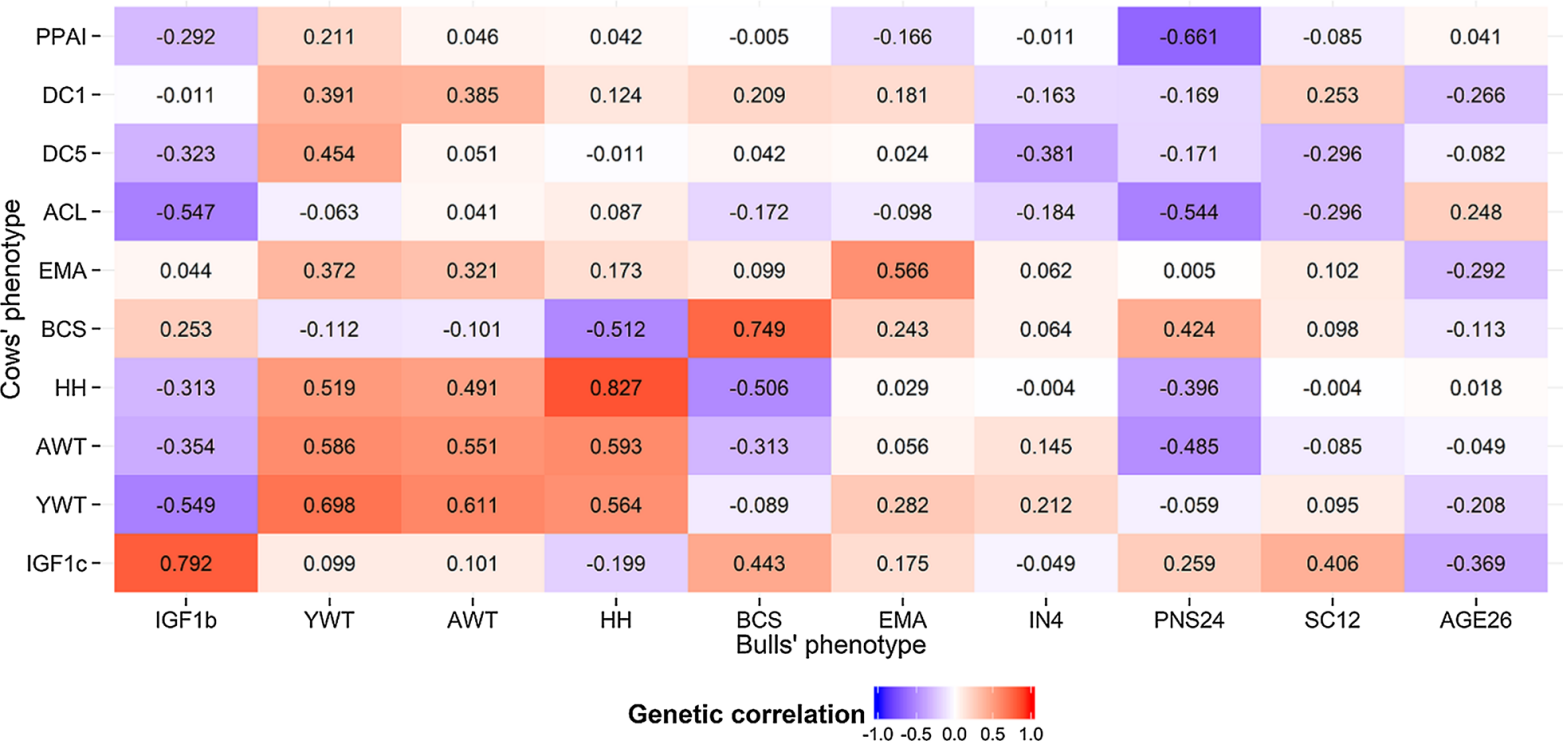
Heat map of the genomic correlations estimated between ten male and ten female growth and reproductive traits (as described in Table 1) in Brahman cattle

Estimated genetic correlations between homologous male and female traits were favorable and ranged from moderate to high values (0.55 ± 0.09 to 0.83 ± 0.06; Fig. 2), this indicates that selection for any of these would lead to a correlated response in the other sex.

The lowest genetic correlations were estimated between AWT in cows and bulls (0.55 ± 0.09), and between EMA in cows and bulls (0.57 ± 0.16) (Fig. 2), which indicate the presence of genotype-by-sex interactions for these traits. The efficiency of the correlated responses between male and female homologous traits was favorable but lower than 1, indicating the superiority of direct over indirect selection [see Additional file 1: Figure S1], except for HH for which selection in cows was estimated to result in 127% of the response obtained by direct selection of HH in bulls. Similarly, selection for HH in cows could be an effective selection criterion for improving additional male growth traits since it was estimated to be associated with 108% and 119% of the response obtained by direct selection of YWT and AWT in bulls, respectively.

Furthermore, Pearson correlations of 0.94, 0.87, 0.74, 0.95, 0.92, and 0.77 were obtained between GEBV of the six male and female homologous IGF1, YWT, AWT, HH, BCS, and EMA phenotypes in the bivariate analyses, respectively [see Additional file 2: Figure S2], and similarly Pearson correlations between GEBV from the analyses for sex-combined traits and for traits separately for each sex ranged from 0.88 to 0.99 [see Additional file 1: Figure S1]. Therefore, in spite of differences between direct and indirect selection responses, small variations in ranking would be expected regardless of whether the GEBV was obtained from the same sex phenotype or the opposite-sex phenotype when both male and female phenotypes are available.

In addition, the six homologous traits allowed us to explore the relative weight of each (male or female) source of information in the resulting GEBV when this GEBV is used to predict the opposite sex. We based these calculations on the correlation of the GEBV of a given trait and sex with the adjusted phenotype for the same or opposite sex. Thus, when exploring the efficiency with which GEBV predict the six homologous traits across sexes, GEBV for one sex was explored against (correlated with) the adjusted phenotype separately for each sex. The ratio between these two correlations reflects the efficiency of predictive power of a GEBV when used to predict the opposite sex. We found that the GEBV benefits for the opposite sex phenotype were around 10% smaller than those obtained from the same sex phenotype (Table 3). For instance, the correlation between the GEBV for IGF1 blood concentration measured in females and the adjusted phenotype for females is 0.48, whereas the correlation between the GEBV for IGF1 blood concentration measured in females and the adjusted phenotype for males is 0.42, i.e. equivalent to 88% of 0.48. Similarly, the correlation between the GEBV for IGF1 blood concentration measured in males and the adjusted phenotype for females is 0.35, which is 95% of the 0.37 correlation observed between the GEBV for IGF1 blood concentration measured in males and the adjusted phenotype for males. The values for efficiency of predictive power reflect the within-sex heritability estimates and the across-sex genetic correlations.

**Table 3:** Relative weight of each source of information (male or female) in the resulting genomic estimated breeding values (GEBV) of homologous male and female traits and efficiency of predictive power in the opposite sex (in parenthesis) when the adjusted phenotypes are measured in the opposed sex only

Traits^a^	Adjusted phenotype from	Correlation with	
		Female GEBV^b^	Male GEBV^b^
IGF1	Female	0.48	0.35 (0.95)
	Male	0.42 (0.88)	0.37
YWT	Female	0.55	0.60 (0.85)
	Male	0.45 (0.82)	0.71
AWT	Female	0.78	0.39 (0.67)
	Male	0.59 (0.76)	0.58
HH	Female	0.97	0.65 (0.89)
	Male	0.92 (0.95)	0.73
BCS	Female	0.87	0.58 (0.85)
	Male	0.79 (0.91)	0.68
EMA	Female	0.76	0.45 (0.76)
	Male	0.55 (0.72)	0.59

^a^Traits are as described in Table 1

^b^Female and male GEBV are of the same dimention and equal to the total number of animals (N = 2111) including 995 females and 1116 males

### Genetic correlations between male and female growth or reproductive traits

Estimated genetic correlations for the same trait in the two sexes were moderately to strongly positive and ranged from 0.55 ± 0.09 for AWT to 0.83 ± 0.06 for HH (Fig. 2 and Additional file 3: Table S1). However, genetic correlations were negative between BCS in one sex and either YWT, AWT or HH in the other sex.

The reproductive traits in males (PNS24, SC12, and AGE26) were favorably associated to ACL (r_g_ = − 0.54 ± 0.11 for ACL and PNS24; − 0.30 ± 0.04 for ACL and SC12 and 0.25 ± 0.08 for ACL and AGE26). The genetic correlation between PNS24 and PPAI was also strong (r_g_ = − 0.66 ± 0.05; Fig. 2). However, selection for reproductive traits in males is associated with only 29% and 20% of the improvement obtained by direct selection of ACL and PPAI, respectively. These results may be partially explained by the *h*^2^ estimates for female being higher than for male reproductive traits.

The standard errors of the genetic correlations between EMA in one sex and growth and reproductive traits in the other sex were high, which indicate that there was no significant association between EMA and the other evaluated traits.

### Genetic correlations between hormone concentrations and male and female growth or reproductive traits

The genetic correlation between IGF1c and male reproductive traits (PNS24, SC12, and AGE26) were favorable and of moderate magnitude (Fig. 2 Additional file 3: Table S1). The genetic correlation and estimated efficiency of the correlated response (ECR) in parenthesis were equal to 0.26 ± 0.03 (0.88) for IGF1c and PNS24; 0.41 ± 0.13 (0.32) for IGF1c and SC12, and − 0.37 ± 0.08 (− 0.25) for IGF1c and AGE26. The genetic correlation between IGF1b and female reproductive traits (ACL and PPAI) were favorable, with ECR of − 0.47 and − 0.35 for ACL and PPAI, respectively. Furthermore, IGF1b was associated to growth traits in females with estimated genetic correlations of − 0.31 ± 0.09 between IGF1b and HH in cows (HHc); − 0.60 ± 0.09 between IGF1b and YWT in cows (YWTc); and 0.35 ± 0.11 between IGF1b and AWT. Estimated genetic correlations of IGF1c or IGF1b with growth traits were either negative or non-significant and ranged from − 0.55 ± 0.09 between IGF1b and YWT in cows, to 0.10 ± 0.13 between IGF1c and AWT in bulls, with an ECR ranging from − 0.64 to 0.09.

The standard errors associated with the estimated genetic correlations between IN4 in bulls and all other traits in cows were high, which indicate that there was no significant genetic correlation between IN4 and the other traits.

### Association between growth and reproductive traits

High standard errors were associated with the estimated genetic correlations between male growth traits and female reproductive traits, which means that the estimated genetic correlations did not significantly differ from zero. However, selection for increased AWTc and HHc was expected to result in lower PNS24 (r_g_ = − 0.49 ± 0.05 and − 0.40 ± 0.04, respectively).

## Discussion

### Heterogeneity of variances and heritability for male and female growth and reproductive traits

Although males and females share close genetic architectures, sexual differences are widespread [18]. In our study, we found differences in the additive and residual variances of homologous traits in males and females, which could be partially explained by a distinct expression of alleles or genes through sexual antagonism [19], distinct mutational effects between males and females [20, 21], presence of sex-specific dominance effects [22], and/or differences in environmental treatments (such as differences in age at measurement of EMA in this study). Rowe and Houle [23] suggested that females are expected to experience stabilizing natural selection on most traits, leading to a reduction in additive variance, while males are expected to experience directional selection on mating-related traits, which could reduce or increase additive variance. In breeding systems, differences in the intensity of selection between sexes could change the additive genetic variance in each sex.

Except for DC1, we found moderate to high *h*^2^ estimates for the evaluated phenotypes, which indicate that these traits can be used in genetic selection programs. For DC1, the *h*^2^ estimates was close to zero, which indicates a greater influence of environmental factors. Moreover, since the dataset included only fertile females and no non-calving females, trait variability and genetic differences between animals may be masked [24], which could partially explain, this low *h*^2^. Similarly to our results, Meyer et al. [25], Johnston and Bunter [26], Mercadante et al. [24] and Mucari et al. [27] reported *h*^2^ estimates for days to calve lower than 0.1. Exploring the correlated response could be more efficient to achieve the highest rates of genetic progress than direct selection for low *h*^2^ traits.

### Across-sex genetic correlations

The across-sex genetic correlations, whether positive or negative, mean that sex-specific homologous traits are not free to evolve independently. It is possible to explore the correlated response when selection is applied in only one sex. However, this correlated response depends on the genetic correlation between the pair of traits and the *h*^2^ estimates for each sex. For instance, if a directional selection is applied for homologous traits in the two sexes, the ability of each sex to reach its optimum will be maximally constrained at r_g_ = 0 and not constrained at r_g_ = 1 [28].

In our study, the presence of genotype-by-sex interactions for EMA and AWT was established based on the heterogeneity of the additive and residual variances and on the r_g_ estimates between homologous traits being lower than the threshold of 0.70 proposed by Mulder et al. [29]. Based on the values of variances, accuracies, genetic correlations and efficiency of correlated response between these homologous traits, we should recommend a sex-specific genetic evaluation, because a given trait measured in each sex cannot be considered as the same trait. However, the strong Pearson correlations between GEBV obtained from same-sex phenotype information and opposite-sex phenotype information as well as the strong Pearson correlations between GEBV from analyses of traits treated separately for each sex and of combined-sex traits indicate that data collection of homologous traits in one sex contributes to estimate GEBV in the other sex. When both male and female phenotypes are included in the analyses, considering male and female phenotypes as a single trait may have no practical impact on selection and ranking of animals.

Most breeding companies assume a genetic correlation of 1 between male and female traits because combined-sex analyses require a simpler model and less computational demand [8]. Similar results are found in the literature i.e. Van Vleck and Cundiff [30] reported an r_g_ between homologous growth traits in males and females higher than 0.85, van der Heide et al. [8] found an r_g_ between male and female post-weaning productive traits that ranged from 0.68 to 0.84, while Raidan et al. [31] obtained an r_g_ of 0.96 for final weight and 0.74 for average daily gain in male and female Nellore cattle raised on pasture. A genetic correlation lower than 1 between male and female traits could be due to limitations of the experimental design and size, or to differences in the performance testing environments between females and males (for instance, differences at age of measurement of EMA in this study), or differences in the genetic bases and/or selection objectives, and means and standard deviations in each sex [32]. A further explanation is the existence of differences between the sex chromosomes which, although not used in the present study, might still harbour differing genetic variation for productive traits in males and females [33].

### Genetic correlation between male and female growth or reproductive traits

We observed high and favorable genetic associations between male and female growth traits, thus, if the purpose is to increase growth traits, those with the higher *h*^2^ estimates could be used as selection criteria because faster genetic changes can be reached by selecting such traits. In this study, the highest *h*^2^ estimate was obtained for HHc, and thus it could be used as a selection criterion for growth traits. Similarly, Regatieri et al. [34] showed positive and moderate genetic correlations estimates of AWT with HH (r_g_ = 0.65 ± 0.01) and concluded that selection for HH may be advantageous in extensive rearing systems since this type of selection is a reliable alternative to control frame size and, consequently, AWT in beef cattle. The monitoring of animal size allows the selection of biotypes that are compatible with the production system since animals with a larger mature size are associated with higher maintenance costs. Previous studies have shown a positive genetic correlation, higher than 0.5, between frame scores and growth traits in beef cattle [35–38]. However, our results showed negative genetic correlations between BCS and YWT and between AWT and HH, which suggest the existence of pleotropic or linked genes that allow growth improvement while maintaining smaller-frame size.

In beef cattle breeding programs, scrotal circumference (SC) is frequently used as a selection criterion to improve male and female reproductive traits [39, 40]. However, it is worth noting that the age at which the traits are recorded has a strong impact on the across-sex correlated response. In taurine breeds, SC recorded at an early age (~ 300 days of age) better reflects female precocity than a later record [41], whereas in tropical breeds that, in general, mature later than taurine cattle, SC12 is a modest genetic predictor of heifer age at puberty [4]. In agreement with Johnston et al. [4], our results showed that the genetic correlations between PNS24, AGE26 and female reproductive traits were stronger than those between SC12 and female reproductive traits, which suggest that PNS24 and AGE26 are better selection criteria for reproduction traits. This can be partly explained by the fact that PNS24 and AGE26 are more strongly associated with puberty in tropical cattle than SC12. In spite of their higher *h*^*2*^ estimates, male and female traits such as PNS24, hormone levels, PPAI and ACL, have been less exploited in breeding schemes [4, 42]. Therefore, measurements in bulls for traits such as PNS24, IGF1, and AGE26, are potentially useful as indirect selection criteria for improving female reproduction in tropical breeds.

### Hormone concentrations (IGF1 and IN4) as selection criteria for male and female growth or reproductive traits

IGF1c level was a satisfactory selection criterion for male reproductive traits, especially PNS24, while IGF1b level showed a favorable association with ACL, YWTc and HHc, and a negative genetic correlation with AWTc. Yilmaz et al. [43] reported that IGF1 level measured in pre-pubertal *Bos taurus* bulls is genetically correlated with adult scrotal circumference, sperm motility, age at first calving and calving rate. These authors confirmed that IGF1 plays an important role in follicular development and ovulation in cattle because IGF1 receptors and estrogen receptors interact to regulate female reproduction and behavior. IGF1 is synthesized in almost all the tissues and has a positive effect on cell proliferation, transformation, and differentiation.

The maternal plasma IGF1 concentration plays an important role in energy balance, average daily gain and nutritional regulation of post-partum reproductive performance in cattle [44]. Miah et al. [45] reported that with the addition of IGF1 in vitro, some spermatozoa characteristics such as progressive motility, induction of capacitation and acrosome reaction have increased, which may partially explain the moderately positive genetic correlation between PNS24 and IGF1c obtained in our study. Furthermore, endocrine IGF1 level acting as a monitoring signal that allows reproductive events to occur when nutritional conditions for successful reproduction are reached [46]. Similarly, IGF1 levels have been shown to be an important regulator of energy metabolism, which may explain the favorable genetic correlation between IGF1b and female growth traits [44].

In males, inhibin is produced mainly by the Sertoli cells and acts in an endocrine manner to negatively regulate the synthesis and release of follicle-stimulating hormone from the anterior pituitary gland [47], which explains the genetic correlation between male reproductive traits and IN4 [42]. Moreover, Corbet et al. [42] showed a moderate genetic correlation of 0.36 ± 0.11 between IGF1 and IN4 measured in males, and Fortes et al. [48] showed that the gene *HELB* is associated with both, IN4 and IGF1b. Cai et al. [47] also observed that, when inhibin A was silenced in Sertoli cells, the expression of IGF1 decreased, which suggests a correlation between these traits. However, we did not find a significant association between IGF1c and IN4.

### Impact of selection for growth traits on reproductive traits

We confirmed the lack of relevant associations between male growth and female reproductive traits previously reported by Meyer et al. [25], Johnston and Bunter [26]; Mercadante et al. [24] and Monteiro et al. [49]. Mercadante et al. [24] reported that a genetic correlation of almost 0 was found between yearling weight in performance-tested young bulls in feedlots (378 days of age) and days to calving of the first mating in Nellore cattle. Later, Monteiro et al. [49] showed that selection for increased yearling weight had no effect on either the development of the ovaries and the endometrium or the onset of puberty at 24 months of age in heifers. To our knowledge, genetic correlations between female growth traits and male reproductive traits have not been reported in the literature; however, our results support the use of AWTc and HHc as selection criteria to improve PNS24.

## Conclusions

Hip height in females is an efficient selection criterion to improve male growth, whereas selection for percentage of normal sperm at 24 months of age and IGF1 blood concentration in bulls at 6 months of age results in only a modest improvement of female reproductive traits. In the presence of genotype-by-sex interactions, within-sex selection is expected to result in high rates of genetic improvement; however, the results of our analyses for traits treated separately for each sex and combined for both sexes showed no practical differences for the identification of animals with extreme breeding values.

## Additional files

**Additional file 1.** **Figure S1**: Efficiency of correlated responses for male (left) and female (right) growth and reproductive traits (as described in Table 1) in Brahman cattle.

**Additional file 2.** **Figure S2**: Pearson correlations between genomic estimated breeding values for cow and bull traits (as described in Table 1) from analyses that treat the traits separately for females and males (BiM and BiF) and that combine them as a single trait (Joined) in Brahman.

**Additional file 3.** **Table S1**: Genomic correlations ± standard deviations estimated between ten male and ten female growth and reproductive traits in Brahman cattle.

## Data Availability

The datasets used and/or analysed during the current study are available from Antonio Reverter on reasonable request.
