# Supplementary figures and images for: Across-sex genomic-assisted genetic correlations for sex-influenced traits in Brahman cattle
Source: Genet Sel Evol. 2019 Jul 23;51:41. doi: 10.1186/s12711-019-0482-6 (PMC6651968; doi:10.1186/s12711-019-0482-6)

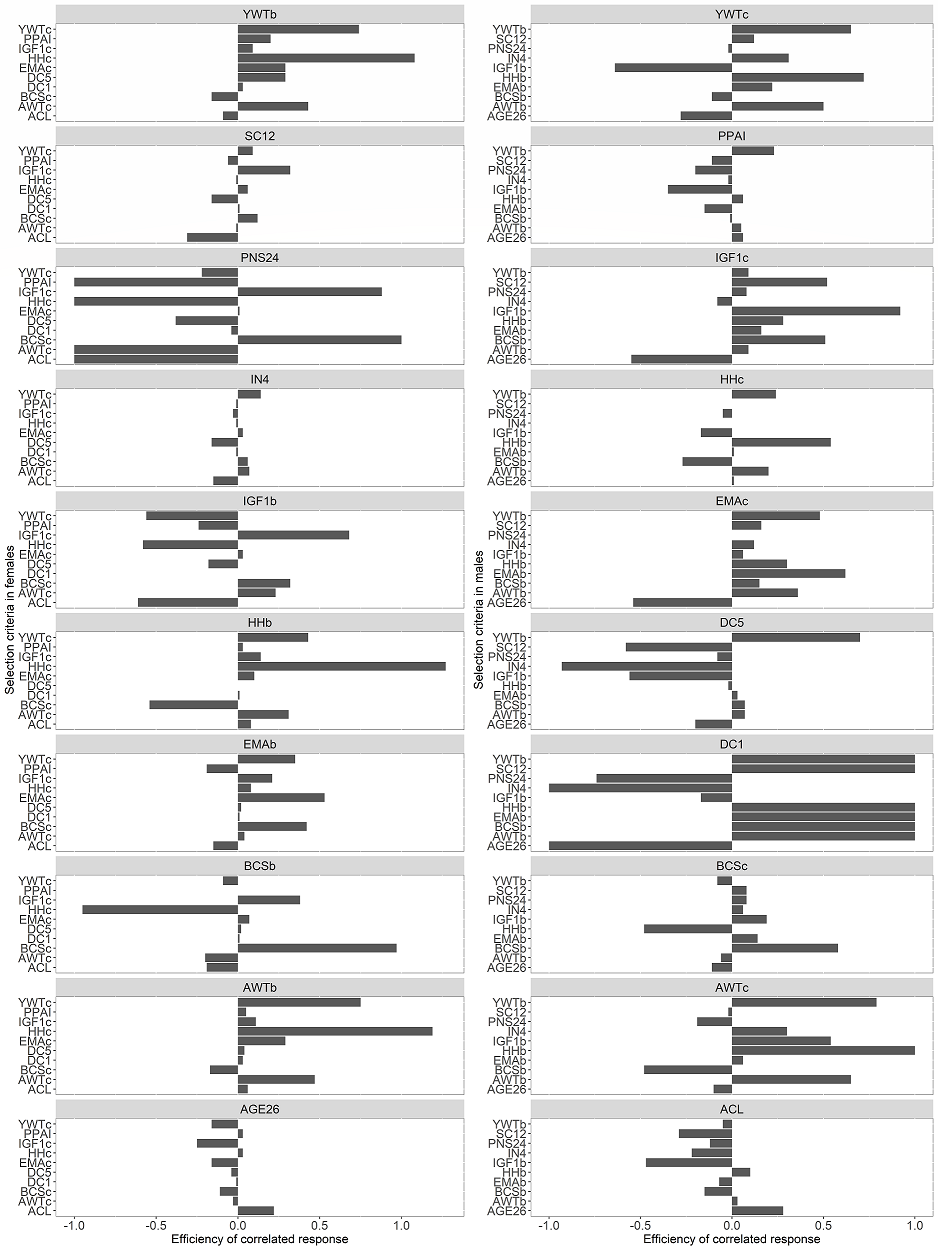

Supplement: Supplementary file 1 — Additional file 1. Figure S1: Efficiency of correlated responses for male (left) and female (right) growth and reproductive traits (as described in Table 1) in Brahman cattle. [file 12711_2019_482_MOESM1_ESM.tiff]

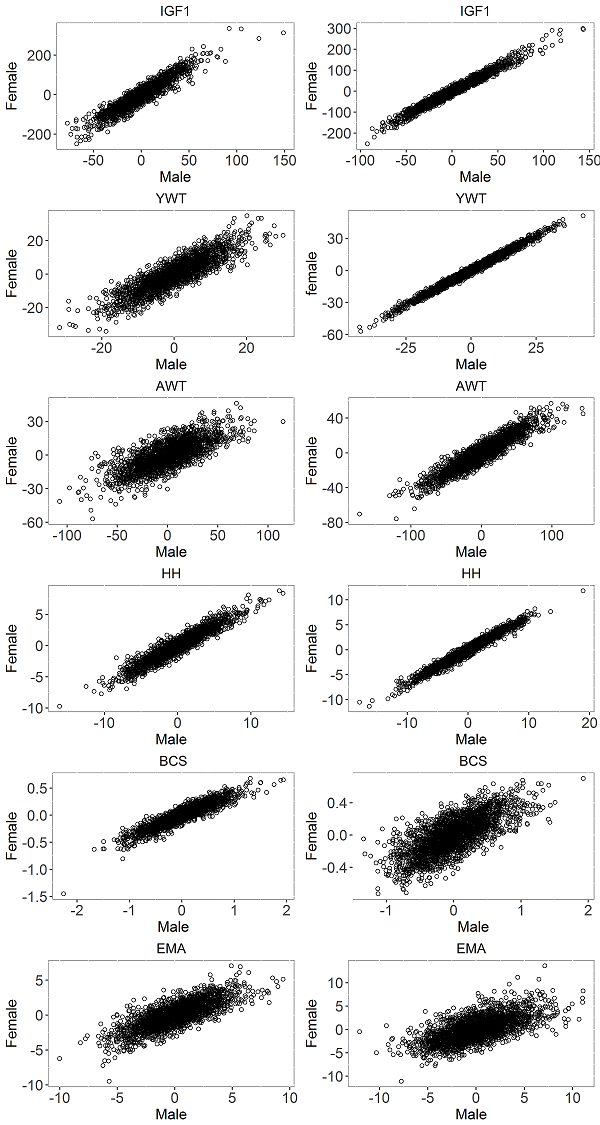

Supplement: Supplementary file 2 — Additional file 2. Figure S2: Pearson correlations between genomic estimated breeding values for cow and bull traits (as described in Table 1) from analyses that treat the traits separately for females and males (BiM and BiF) and that combine them as a single trait (Joined) in Brahman. [file 12711_2019_482_MOESM2_ESM.tiff]
