# Supplementary material for: Across-sex genomic-assisted genetic correlations for sex-influenced traits in Brahman cattle
Source: Genet Sel Evol. 2019 Jul 23;51:41. doi: 10.1186/s12711-019-0482-6 (PMC6651968; doi:10.1186/s12711-019-0482-6)
Supplement: Supplementary file 3 — Additional file 3. Table S1: Genomic correlations ± standard deviations estimated between ten male and ten female growth and reproductive traits in Brahman cattle. [file 12711_2019_482_MOESM3_ESM.docx]

**Table S1. Genomic correlations ± standard deviations estimated between ten male and ten female productive and reproductive traits in Brahman cattle**

| **Traits^a^** | **IGF1_B_** | **YWT_B_** | **AWT_B_** | **HH_B_** | **BCS_B_** | **EMA_B_** | **IN4** | **PNS24** | **SC12** | **AGE26** |
| --- | --- | --- | --- | --- | --- | --- | --- | --- | --- | --- |
| PPAI | -0.29±0.15 | 0.21±0.17 | 0.05±0.17 | 0.04±0.15 | -0.01±0.16 | -0.17±0.19 | -0.01±0.16 | -0.66±0.05 | -0.08±0.16 | 0.04±0.15 |
| DC1 | -0.01±0.19 | 0.39±0.19 | 0.38±0.20 | 0.12±0.18 | 0.21±0.20 | 0.18±0.22 | -0.16±0.22 | -0.17±0.64 | 0.25±0.18 | -0.27±0.18 |
| DC5 | -0.32±0.78 | 0.45±0.65 | 0.05±0.77 | -0.01±0.49 | 0.04±0.65 | 0.02±0.76 | -0.38±0.52 | -0.17±0.54 | -0.30±0.61 | -0.09±0.62 |
| ACL | -0.55±0.11 | -0.06±0.13 | 0.04±0.11 | 0.09±0.11 | -0.17±0.12 | -0.10±0.09 | -0.18±0.11 | -0.54±0.11 | -0.30±0.04 | 0.25±0.08 |
| EMA_C_ | 0.04±0.15 | 0.37±0.16 | 0.32±0.06 | 0.17±0.14 | 0.10±0.16 | 0.57±0.16 | 0.06±0.15 | 0.01±0.32 | 0.10±0.015 | -0.29±005 |
| BCS_C_ | 0.25±0.11 | -0.11±0.11 | -0.10±0.09 | -0.51±0.11 | 0.75±0.10 | 0.24±0.13 | 0.06±0.11 | 0.42±0.04 | 0.10±0.11 | -0.11±0.10 |
| HH_C_ | -0.31±0.09 | 0.51±0.09 | 0.49±0.08 | 0.83±0.06 | -0.51±0.09 | 0.03±0.11 | -0.01±0.09 | -0.40±0.04 | -0.01±0.09 | 0.02±0.09 |
| AWT_C_ | 0.35±0.11 | 0.58±0.10 | 0.55±0.09 | 0.59±0.09 | -0.31±0.11 | 0.05±0.13 | 0.14±0.11 | -0.49±0.05 | -0.01±0.11 | -0.05±0.10 |
| YWT_C_ | -0.55±0.09 | 0.69±0.13 | 0.61±0.11 | 0.56±0.11 | 0.10±0.14 | 0.28±0.15 | 0.21±0.13 | -0.06±0.31 | 0.10±0.12 | -0.21±0.12 |
| IGF1_C_ | 0.79±0.10 | 0.09±0.14 | 0.10±0.13 | -0.19±0.12 | 0.44±0.13 | 0.18±0.16 | -0.05±0.13 | 0.26±0.03 | 0.41±0.13 | -0.37±0.08 |

^a^Traits are as described in Table 1.
